# P-104. Safety and tolerability of SYN-004 in allogeneic hematopoietic cell transplant (HCT) recipients receiving meropenem (MER) or piperacillin/tazobactam (P/T)

**DOI:** 10.1093/ofid/ofaf695.333

**Published:** 2026-01-11

**Authors:** Mark Schroeder, Hailey Sappington, Gabbie Lampen, Kimberly Reske, Tiffany Hink, Victoria Joachimstaler, Jeremy Eisele, Matthew R Keller, Andrew Bristol, Charles Le, Sheila Connelly, Michael Moenk, Vince Wacher, Michael Kaleko, Joseph L Kuti, Margaret A A Olsen, Erik R Dubberke

**Affiliations:** Washington University School of Medicine, Saint Louis, Missouri; Washington University School of Medicine, Saint Louis, Missouri; Washington University School of Medicine, Saint Louis, Missouri; Washington University, St. Louis, Missouri; Washington University, St. Louis, Missouri; Washington University School of Medicine, Saint Louis, Missouri; Washington University in St. Louis, Saint Louis, Missouri; Washington University in St. Louis, Saint Louis, Missouri; Theriva Biologics, Rockville, Maryland; Theriva Biologics, Rockville, Maryland; Thervia Biologics, Rockville, Maryland; Charles River Laboratories, Skokie, Illinois; Theriva Biologics, Rockville, Maryland; Theriva Biologics, Rockville, Maryland; Hartford Hospital, Hartford, CT; Washington University School of Medicine, Saint Louis, Missouri; Washington University School of Medicine, Saint Louis, Missouri

## Abstract

**Background:**

SYN-004 is an oral beta-lactamase enzyme designed to preserve the intestinal microbiome in patients receiving IV beta-lactam antibiotics. Data suggest improved outcomes among HCT recipients with a healthier microbiome at engraftment. This phase 1b/2a trial evaluates the safety, tolerability, and potential absorption of SYN-004 in HCT recipients, divided into three cohorts (MER, P/T, and cefepime). Interim blinded results of the MER and P/T cohorts are presented.

**Table 1.**
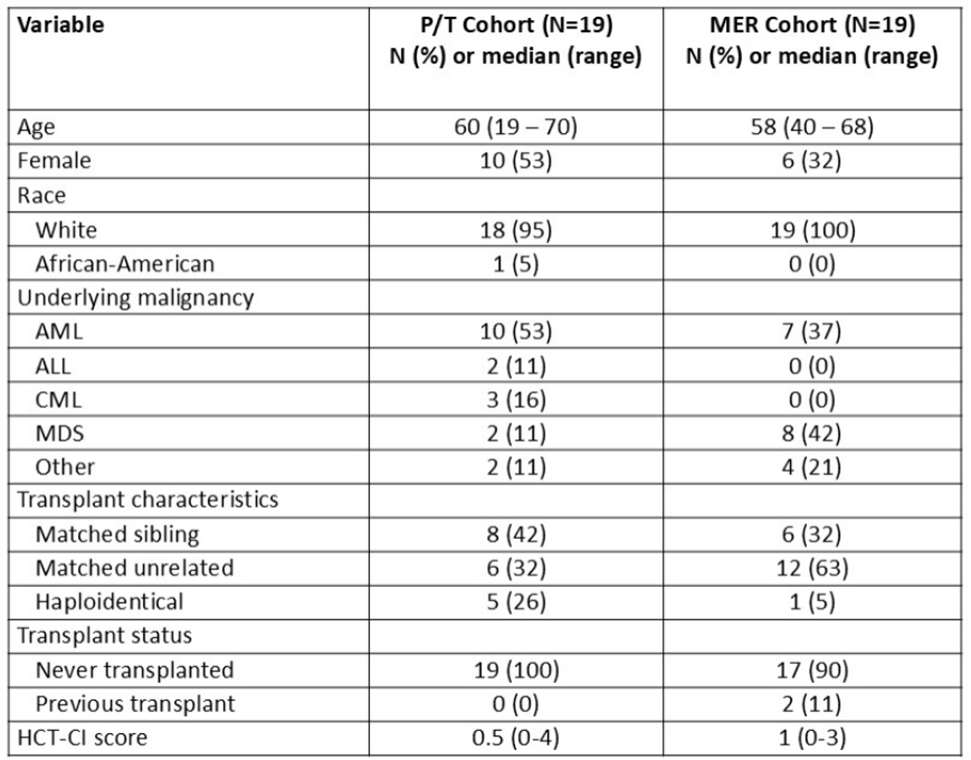
Demographics of the study population (N=38)

**Table 2.**
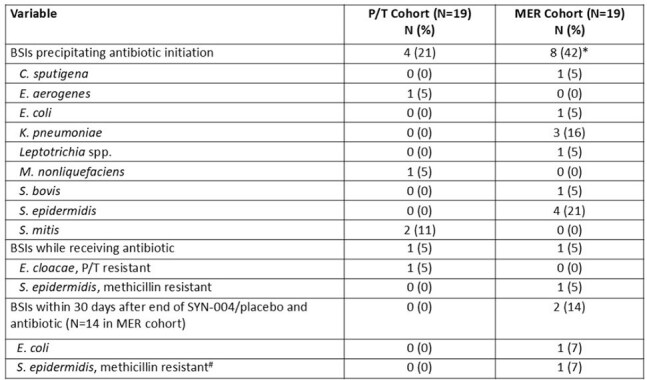
Bloodstream infections among patients who received SYN-004 or placebo (N=38) * Three BSIs were polymicrobial, so total organisms adds up to more than 8. Polymicrobial BSIs included: S. epidermidis / K. pneumoniae; K. pneumoniae / S. bovis; S. epidermidis / C. sputigena. One patient had two separate BSIs: S. epidermidis and then E. coli. #Case of endocarditis reported as SAE.

**Methods:**

Adults undergoing allogenic HCT at Barnes-Jewish Hospital were randomized 2(SYN-004):1(placebo) on HCT day+1 and continued until antibiotic discontinuation. The treating clinicians initiated MER or P/T. Data are presented through 30 days post-study drug discontinuation. Blood for SYN-004 detection was collected at enrollment and < 72 hours after first study drug dose. SYN-004 and antibiotic concentrations were measured at day 2-3 after start and every 7 days after. Target enrollment was 12 participants per cohort completing ≥2 antibiotic PK blood draws. SYN-004 samples were first screened with an electrochemiluminescence immunoassay (ECL) (lower limit of detection (LLD)=0.8ng/ml). Blood positive by ECL was tested with a functional assay (LLD=5ng/ml). Antibiotic exposure was evaluated by population PK (Pmetrics). Grade 3-5 severe adverse events (SAEs) and relatedness to study drug were assessed.

**Table 3.**
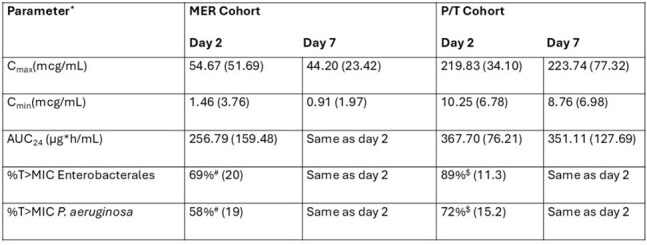
Antibiotic PK for study participants who met evaluable criteria *Results presented as mean (standard deviation) total drug concentrations (Cmax, Cmin, AUCτ) or drug exposures (T>MIC). #Simulated exposure at the Enterobacterales susceptibility breakpoint (1µg/mL) and P. aeruginosa breakpoint (2µg/mL); Target T>MIC is 40%. $Simulated exposure at the Enterobacterales susceptibility breakpoint (8µg/mL) and P. aeruginosa breakpoint (15µg/mL); Target T>MIC is 50%.

**Table 4.**
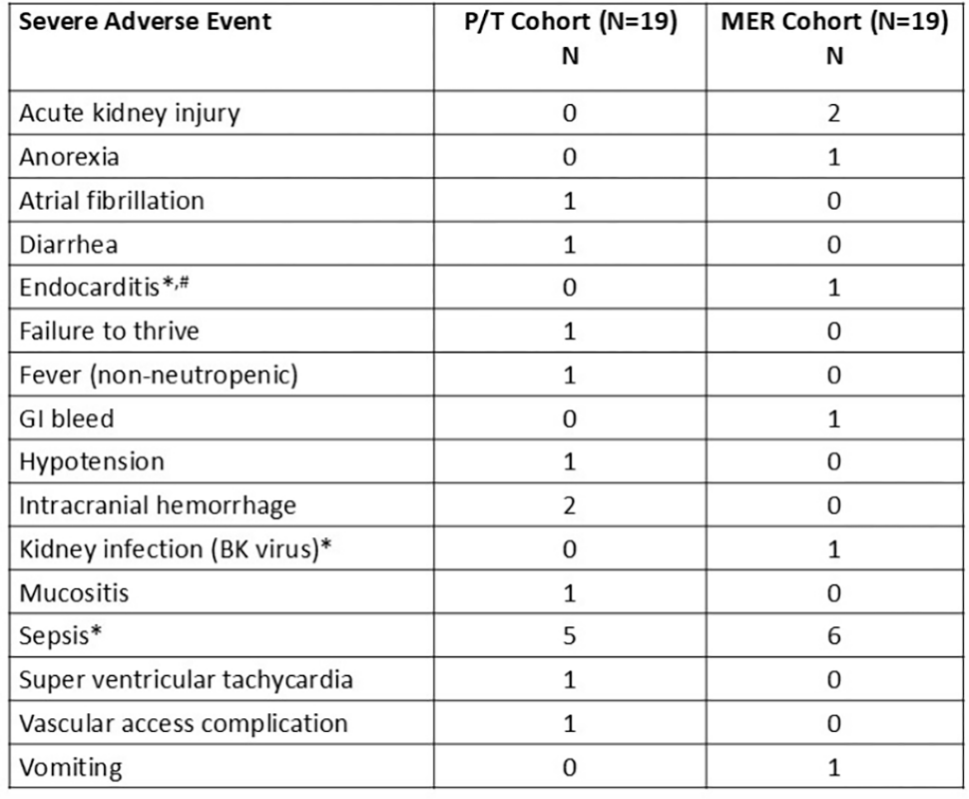
Severe adverse events among study participants (N=38); no SAEs were attributed to study drug *Infectious SAE; one sepsis SAE resulted in death (not related to study drug). #S. epidermidis; occurred after completion of study drug/placebo and meropenem.

**Results:**

19 participants started study drug in each cohort (Table 1); 12 per cohort had ≥2 antibiotic PK periods. 14/38 (37%) developed a bloodstream infection (BSI) while on study drug (Table 2); 12 BSIs were the precipitating event for antibiotic initiation. SYN-004 was detected in 6/233 (2.6%) of blood specimens by ECL. Active SYN-004 was not detected by the functional assay. The mean (SD) area under the curve for MER on day 2 was 257mg*h/L (159); for P/T, it was 368 mg*h/L (76) (Table 3). 28 SAEs occurred; none related to study drug (Table 4).

**Conclusion:**

Interim blinded analyses after the MER and P/T cohorts suggest SYN-004 is well-tolerated in HCT patients. Systemic SYN-004 detection in blood was uncommon by ECL, and functional, active drug was not detected. This study has received approval from the data safety and monitoring committee to proceed to the cefepime cohort.

**Disclosures:**

Andrew Bristol, PhD, Theriva Biologics: Employee|Theriva Biologics: Stocks/Bonds (Public Company) Charles Le, PhD, Theriva Biologics: Employee|Theriva Biologics: Stocks/Bonds (Public Company) Sheila Connelly, PhD, Theriva Biologics: Employee|Theriva Biologics: Stocks/Bonds (Public Company) Vince Wacher, PhD, Theriva Biologics: Contracted to Theriva Biologics and holds Theriva shares|Theriva Biologics: Stocks/Bonds (Public Company) Michael Kaleko, MD, PhD, Theriva Biologics: Employee|Theriva Biologics: Stocks/Bonds (Public Company) Margaret A A. Olsen, PhD, MPH, Ferring Pharmaceuticals: Advisor/Consultant|Pfizer: Advisor/Consultant Erik R. Dubberke, MD, MSPH, AstraZeneca: Advisor/Consultant|AstraZeneca: Grant/Research Support|Merck: Grant/Research Support|Pfizer: Advisor/Consultant|Pfizer: Grant/Research Support|Theriva Biologics: Grant/Research Support|Vedanta: Advisor/Consultant|Vedanta: Grant/Research Support

